# The new Internal Transcribed Spacer 2 diagnostic tool clarifies the taxonomic position and geographic distribution of the North American malaria vector *Anopheles punctipennis*

**DOI:** 10.1186/s12936-021-03676-4

**Published:** 2021-03-10

**Authors:** James M. Hodge, Andrey A. Yurchenko, Dmitriy A. Karagodin, Reem A. Masri, Ryan C. Smith, Mikhail I. Gordeev, Maria V. Sharakhova

**Affiliations:** 1 Department of Entomology and the Fralin Life Sciences Institute, Virginia Polytechnic Institute and State University, Blacksburg, VA USA; 2 Laboratory of Evolutionary Genomics of Insects, the Federal Research Center Institute of Cytology and Genetics, Siberian Branch of the Russian Academy of Sciences, Novosibirsk, Russia; 3 Kurchatov Genomics Center, the Federal Research Center Institute of Cytology and Genetics, Siberian Branch of the Russian Academy of Science, Novosibirsk, Russia; 4 Department of Entomology, Iowa State University, Ames, IA USA; 5 Department of General Biology and Ecology, Moscow Region State University, Moscow, Russia

**Keywords:** *Anopheles punctipennis*, Mosquito, Molecular diagnostics, Internal transcribed spacer, Restriction fragment length polymorphism

## Abstract

**Background:**

The malaria mosquito *Anopheles punctipennis*, a widely distributed species in North America, is capable of transmitting human malaria and is actively involved in the transmission of the ungulate malaria parasite *Plasmodium odocoilei*. However, molecular diagnostic tools based on Internal Transcribed Spacer 2 (ITS2) of ribosomal DNA are lacking for this species. *Anopheles punctipennis* is a former member of the *Anopheles maculipennis* complex but its systematic position remains unclear.

**Methods:**

In this study, ITS2 sequences were obtained from 276 *An. punctipennis* specimens collected in the eastern and midwestern United States and a simple and robust Restriction Fragment Length Polymorphism approach for species identification was developed. The maximum-likelihood phylogenetic tree was constructed based on ITS2 sequences available through this study and from GenBank for 20 species of *Anopheles.*

**Results:**

The analysis demonstrated a consistent ITS2 sequence length and showed no indications of intragenomic variation among the samples based on ITS2, suggesting that *An. punctipennis* represents a single species in the studied geographic locations. In this study, *An. punctipennis* was found in urban, rural, and forest settings, suggesting its potential broad role in pathogen transmission. Phylogeny based on ITS2 sequence comparison demonstrated the close relationship of this species with other members of the Maculipennis group.

**Conclusions:**

This study developed molecular tools based on ITS2 sequences for the malaria vector *An. punctipennis* and clarified the phylogenetic position of the species within the Maculipennis group.

## Background

Malaria is one of the most dangerous infectious diseases transmitted by mosquitoes [1]. In 2018, the World Health Organization (WHO) reported 228 million cases of malaria occurring worldwide. However, in contrast to the African region, where the WHO reports a steady declining of the disease, the rise in malaria cases has been recorded in the American region [2]. Malaria had been a significant problem in North America where it was introduced in the seventeenth century during the slave trade and, because of the presence of native competent malaria vectors, it rapidly spread through the entire continent [3]. In the USA, numerous outbreaks of malaria occurred from as far north as Massachusetts to subtropical southern states in the eighteenth and nineteenth centuries. Starting in the 1930s, intensive efforts to control malaria were undertaken and, in 1954, malaria was declared eliminated from the USA [3]. Nevertheless, each year, the Center for Disease Control and Prevention (CDC) reports between 1,000 and 1,500 cases of malaria in the USA [4], with 63 outbreaks of locally transmitted malaria occurring since 1957 [5]. Climate change, human migration, political instability, and the presence of competent malaria vectors increase the risk of malaria and other tropical diseases being imported and transmitted in regions where they were eradicated [5].

Three species are considered dominant malaria vectors in North America—*Anopheles freeborni*, *Anopheles pseudopunctipennis,* and *Anopheles quadrimaculatus* [6]. In addition to these species, *Anopheles punctipennis*, the most common and widely distributed anopheline mosquito species in North America [7], was an important contributor to malaria transmission in the USA in the past [8]. Similar to *An. quadrimaculatus,* this species is capable of transmitting two major human malaria parasites, *Plasmodium vivax* and *Plasmodium falciparum* [9]. Moreover, *An. punctipennis* can also transmit *Plasmodium odocoilei,* which infects wild hooved animals, such as deer. A recent study conducted in 17 states across the USA estimated a 25% *P. odocoilei* infection rate in white-tailed deer [10]. Malaria in wildlife populations may also have an impact on biodiversity, as infection reduces the deer population by killing fawns [11]. This can also present problems for humans due to the possibility of zoonotic transmission to livestock ungulates vectored from wild ungulates [11]. The ability of *An. punctipennis* to transmit arboviruses, such as Cache Valley [12] and eastern equine encephalomyelitis [13] viruses, as well as a nematode *Dirofilaria immitis* [14] has also been reported.

Continuous study of mosquitoes in the field requires rapid and robust identification of the species. The availability of PCR-based molecular technologies enable the use of ribosomal DNA (rDNA) for species diagnostics [15]. rDNA genes are organized in arrays of multiple copies in eukaryotic genomes and undergo “concerted evolution” which leads to unification of gene and intergenic sequences [16]. Unlike Intragenic Spacer and Intergenic Transcribed Spacer 1, Intergenic Transcribed Spacer 2 (ITS2) is a reliable tool for species diagnostics in the *Anopheles* genus [15]. ITS2 sequences have been utilized for species diagnostics in the *Anopheles gambiae* complex [17], the *Anopheles funestus* group [18], and the *Anopheles bancroftii* group [19]. One nucleotide difference in ITS2 led to the discovery of the M and S forms of *An. gambiae*, which have now been elevated to species status as *An. gambiae* (former S form) and *Anopheles coluzzii* (former M form) [20]. Three new Eurasian species*—Anopheles artemievi* [21], *Anopheles persiensis* [22], and *Anopheles daciae* [23]*—*were described based on differences in their ITS2 sequences. ITS2 sequences have been successfully used for the identification of North American mosquitoes, including a newly identified species, *Anopheles hermsi* [24], and four new species in the Quadrimaculatus complex [25]. More recently ITS2-based diagnostic tools were developed for species from the *Anopheles crucians* complex [26]. Despite the wide distribution in North America and its obvious role in pathogen transmission, a molecular diagnostic tool using ITS2 of the rDNA from *An. punctipennis* has not been developed yet.

In the past, *An. punctipennis* was a member of the former *Anopheles maculipennis* complex, which included 7 Eurasian and 6 North American species [27]. Although, considered distance by the classical morphological approach, *An. punctipennis* was still placed within the *An. maculipennis* complex based on interspecies hybridization with other members of the complex and the structure of its polytene chromosomes [28]. In 2004, Harbach proposed a new classification for the genus *Anopheles* that was largely based on morphological characteristics and ITS2 sequence analyses [29]. According to this classification, the *An. maculipennis* complex was transformed to the Maculipennis group, which was subdivided into two North American subgroups, Freeborni and Quadrimaculatus, and one Eurasian Maculipennis subgroup. *Anopheles punctipennis* was excluded from the Maculipennis group and placed into a separate Punctipennis group that included *An. punctipennis, Anopheles perplexens,* and the *An. crucians* complex.

In this study, malaria mosquitoes were collected from 10 sites in eastern (Virginia and Florida) and midwestern states (Iowa and Minnesota) and 2 assays based on the sequencing of ITS2 of the rDNA and Restriction Fragment Length Polymorphism (RFLP) of ITS2 for molecular diagnostics of *An. punctipennis* were developed. In addition, ITS2 sequences were used to clarify the phylogenetic relationships of *An. punctipennis.*

## Methods

### Mosquito collection

**Fig. 1:**
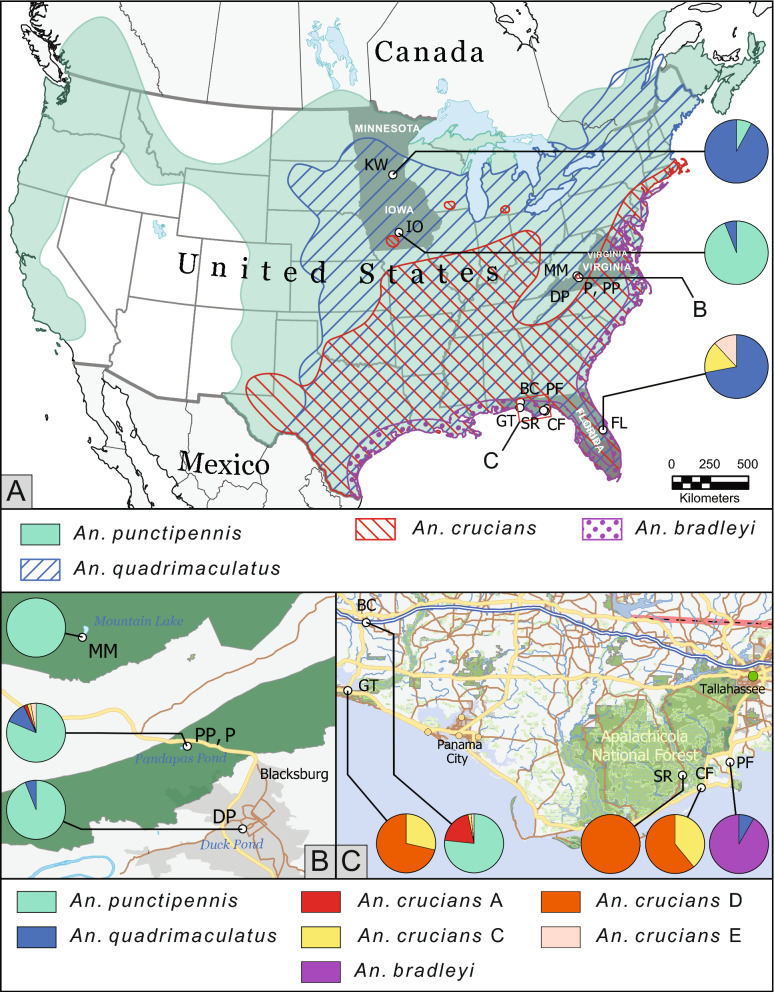
Species composition in mosquito collections in eastern and midwestern United States. Species distribution [35] is shown on the map by different shades (**a**). The map was developed using the program ArcGis Pro [30]. Species abundance is shown by different colors in pie charts. Details of the site locations and species compositions in Virginia and Florida are shown in panels B and C, respectively. Two letter code represents the following: KW stands for Minnetrista, Minnesota; IO for Des Moines, Iowa; DP for Blacksburg, Virginia; P, PP for Montgomery County, Virginia; MM for Giles County, Virginia; BC for Defuniak Springs, Florida; PF for Panacea, Florida, CF for Carrebelle, Florida; SR for Apalachicola, Florida; GT for Greenway Trail, Florida, FL for Melbourne, Florida. *An. punctipennis* was found in all locations except for Costal Florida

A collection of approximately 500 Anopheles mosquitoes were sampled from multiple locations across four states during the summers of 2017 and 2018 as shown in Table 1. The collections from Iowa were provided by the colleagues from Iowa State University. Geographical positions of the mosquito collection sites are shown on a map using the program ArcGis Pro [30] in Fig. 1. Mosquitoes were collected from larval breeding sites, typically small ponds or bodies of water. These breeding sites were located near aquatic plants known as the filamentous algae. More specifically the plants among these breeding sites were identified as *Cladophora* sp.*, Spirogyra* sp*.,* and *Pithophora* sp*.* The pan dipping method was used to collect samples of *Anopheles* in their natural habitat. A pan was dipped under the water so that the surface water spilled into the pan. Once in the pan, a disposable pipette was implemented to draw the larvae from the water, which was then placed into a collection container. 50 ml centrifuge tubes were used for temporary storage while in the field. After that, the larvae were fixed in 70% ethanol. The mosquito larvae were individually labelled for identification and placed in 1.5 ml tubes and stored at − 20 °C.

**Table 1:** Mosquito collections from the eastern and midwestern United States

State	County	Site location (two letter code)	Latitude	Longitude	Number	*An. punctipennis*	*An. quadrimaculatus s.s*	*An. bradleyi*	*An. crucians A*	*An. crucians C*	*An. crucians D*	*An. crucians E*
Minnesota	Hennepin	Minnetrista (KW)	44,95,972	− 93,709,693	38	3	35	0	0	0	0	0
Iowa	Polk	Des Moines (IO)	41,53,825	− 93,57,757	50	47	3	0	0	0	0	0
Florida	Wakulla	Panacea (PF)	30,0347	− 84,3894	12	0	1	11	0	0	0	0
	Walton	DeFuniak Springs (BC)	30,6887	− 86,0945	60	46	0	0	12	1	0	1
		Greenway Trail (GT)	30,3708	− 86,1807	49	0	0	0	0	14	35	0
	Franklin	Carrabelle (CF)	29,9151	− 84,524	46	0	0	0	0	18	28	0
		Appalachicola (SR)	29,973,512	− 84,61,206	12	0	0	0	0	0	12	0
	Osceola	Melbourne (FL)	28,136	− 80,897	25	0	18	0	0	4	0	3
Virginia	Montgomery	Pandapas Pond (P,PP)	37,282,164	-80,465,947	102	83	12	0	2	2	0	3
		Duck Pond (DP)	37,22,592	− 80,427,208	50	47	3	0	0	0	0	0
	Giles	Mountain Lake (MM)	37,356,167	− 80,536,283	50	50	0	0	0	0	0	0
Total					494	276	72	11	14	39	75	7

### DNA extraction

DNA extractions were conducted from mosquito larvae, except at the location in Iowa, where DNA were extracted from adults. DNA extractions were attempted for ~ 500 specimens. Each individual carcass was placed in a sterile 1.5 ml tube to prevent contamination. Extraction of genomic DNA from each specimen was conducted using the standard protocol for Qiagen DNeasy Blood and Tissue Kits (Qiagen, Germantown, MD, USA) with some modification. DNA elution was performed with 50 μl of molecular grade water (Research Products International, Mt. Prospect, IL, USA). Purified DNA was checked using a Nanodrop spectrometer (Thermo Fisher Scientific, Haverhill, MA, USA) to determine quantity and quality and then stored at − 20 °C.

### DNA amplification

For rDNA ITS2 amplification, the direct primer from Djadid et al*.* [31] with some modification 5′-ATCACTCGGCTCTCGTGGATCG-3′ and the reverse primer design by Proft et al*.* 5′-ATGCTTAAATTTAGGGGGTA-3′ were used [32]. For the PCR reaction, Hot Start ImmoMix™ (Bioline, Taunton, MA, USA) was used. PCR reaction mixtures were conducted using 3 μl of genomic DNA, 15 μl 2 × ImmoMix, 1 μl of both forward and reverse primers at 10 µM concentration, and 10 μl of molecular grade water. PCR amplification was conducted using a thermal cycler (Eppendorf, Hauppauge, NY, USA) with the following parameters: 95 °C for 10 min, followed by 30 cycles of 94 °C for 15 s, 55 °C for 30 s, and 72 °C for 30 s, and the final step at 72 °C for 5 min.

The mitochondrial cytochrome c oxidase subunit I (COI) gene was amplified using the following primers that were designed using Primer 3 software: Forward 5′-TGGAGCCTTTATTGGAGACG—3′ and Reverse 5′-ATAGTAGAAAATGGGGCCGG—3′. PCR amplification was conducted with the following parameters for COI: 95 °C for 10 min, followed by 30 cycles of 94 °C for 30 s, 55 °C for 30 s, 72 °C for 30 s, and a final step at 72 °C for 7 min. PCR products were analysed by electrophoresis in 1% agarose gel. GelRed® Nucleic Acid Gel Stain (Biotium, Fremont, CA, USA) was used to visualize the bands. For DNA sequencing, amplicons were purified with a Wizard™ PCR Clean Up kit (Promega, Fitchburg, WI, USA). The product was stored at − 20 °C until processed for sequencing.

### DNA sequencing and species diagnostics

10 µl of PCR products were mixed with 3 μl forward or reverse primers at 3.2 μM concentration and followed be a standard Sanger sequencing procedure [33]. The raw sequences obtained from local collections were uploaded to Sequencher 5.3 software (Gene Codes Corp. Ann Arbor, MI) for further analysis. Species were identified by comparing the obtained sequences with sequences available in GenBank using NCBI BLAST [34]. Because ITS2 sequences were unavailable for *An. punctipennis* at the time when the research was conducted this species was identified using additional sequencing of the COI gene; samples were identified as *An. punctipennis* if they had ≥ 99% identity with *An. punctipennis* COI sequences from GenBank [30]. This comparison allowed to match the ITS2 sequence with the COI sequence from the same individual. In addition, samples from Iowa were analysed morphologically as *An. punctipennis* at the adult stage [35].

Sequences were analysed for quality and for 114 specimens sequenced from both ends the forward and reverse sequences were assembled to obtain the complete sequences of ITS2. Resulting ITS2 nucleotide sequence of *An. punctipennis* (MW581372-MW581485) and a consensus sequence (LR877261) were submitted to the GenBank [30].

### DNA restriction

To obtain DNA fragments with different lengths for *An. punctipennis*, 4 μl of the ITS2 PCR product, 2 μl of the 10 × CutSmart® Buffer, 5 units (0.5 µl) of NaeI restriction enzyme (New England BioLabs, Inc., Ipswich, MA, USA**),** and 18 μl of ddH_2_O were added to a sterile PCR tube. The tubes were then placed in a thermocycler at 37 °C for 60 min. PCR products were analysed by electrophoresis in 3% agarose gel. GelRed® Nucleic Acid Gel Stain was used to visualize the bands.

### Phylogenetic analysis

ITS2 sequences representing 20 Anopheline species were downloaded from the GenBank [34]. The sequences were aligned using the webPRANK server [36] and then the model of nucleotide substitutions was estimated using jModelTest 2 software [37] and Bayesian information criterion. The maximum-likelihood tree was constructed using MEGA X [38] software with the HKY-G model and tested using 500 bootstrap-replications. All sites with gaps or missing data were excluded from the alignment during tree construction.

## Results

### Internal Transcribed Spacer 2 sequencing

The primary goal of this study was to develop a molecular tool based on ITS2 for the identification of *An. punctipennis*. To accomplish this goal, mosquitoes were collected from multiple locations in several eastern and midwestern states in the USA (Table 1, Fig. 1). A total of 494 *Anopheles* samples, which included 276 *An. punctipennis* samples, were successfully sequenced. ITS2 sequences of sampled individuals of *An. punctipennis* from various locations showed no intraspecific differences among the samples tested. All of the samples had a consistent sequence length and there was no indication of interindividual variation among them based on ITS2. The sequences of ITS2 for 114 samples, which were sequenced from both ends were submitted to the GenBank [34].

### Restriction Fragment Length Polymorphism approach

**Fig. 2:**
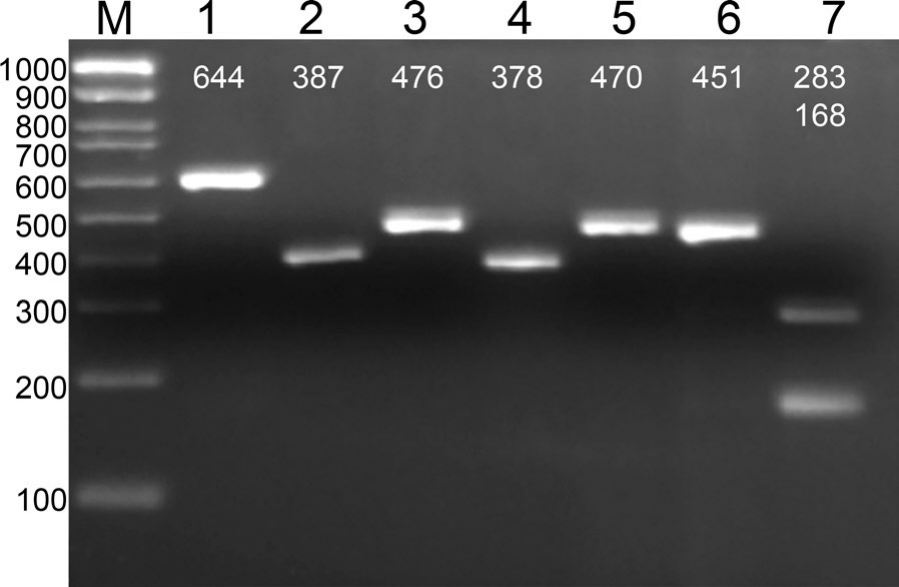
Restriction fragment length polymorphism approach for detecting *An. punctipennis.* Results of gel electrophoresis of the ITS2 PCR product digested with restriction enzyme Nael is shown for the following species: (1) *An. crucians* A (644 bp); (2) *An. crucians* C (387 bp); (3) *An. crucians* D (476 bp); (4) *An. crucians* E (378 bp); ; (5) *An. quadrimaculatus* (470 bp); (6) *An. punctipennis*, undigested; and (7) *An. punctipennis*, digested (283 bp and 168 bp). Column M represents a 1 kb DNA ladder (Millapore Sigma, Sigma Aldrich USA). Only the *An. punctipennis* ITS2 PCR product was cleaved by the enzyme resulting in the appearance of two bands

In addition to ITS2 sequencing, a Restriction Fragment Length Polymorphism (RFLP) approach was developed for the rapid identification of *An. punctipennis* among the other species that can be simultaneously present in the same field collections. The sizes of PCR products of ITS2 for the species that were obtained in the mosquito collections were the following: *Anopheles bradleyi* 391 bp, *An. crucians* A 644 bp, *An. crucians* C 387 bp, *An.* *crucians* D 476 bp, *An. crucians* E 378 bp, *An. punctipennis* 451 bp; and *An. quadrimaculatus* 470 bp. Thus, based on PCR product length, it is hard to distinguish *An. punctipennis* from *An. quadrimaculatus* and *An.* *crucians D* (Fig. 2). Based on the restriction enzyme map of ITS2 sequences, a NaeI restriction enzyme was chosen that cleaves the PCR product of *An. punctipennis,* resulting in the appearance of two fragments of 168 bp and 283 bp. There are no sites for this restriction enzyme in the ITS2 sequences in any of the other species that were found together in all mosquito collections (Table 1). This approach was tested on 50 samples from the various sample locations: 9 samples each from the Defuniak Springs location in Florida and in the Des Moines location in Iowa, and 3 samples from the Minnetrista location in Minnesota. The overall analysis of the 50 samples of *An. punctipennis* evaluated by the RFLP-ITS2 assay indicated that 100% of the samples displayed a double band. The assays with all the collected anopheline species determined that *An. punctipennis* was the only species that displays a double band result, indicating high reliability of this approach the identification of *An. punctipennis* (Fig. 2). In addition to the species collected in this study, the ITS2 sequences from the other 704 species from genus *Anopheles*, were also analysed using data from GenBank [34]. Of 55 species with NaeI site, only six species were from North America: *Anopheles aquasalis, An. crucians B*, *Anopheles darlingi, Anopheles neivai*, *Anopheles punctimacula,* and *An. pseudopunctipennis*. However, most of these species can be easily distinguished from *An. punctipennis* by the size of the PCR products and restriction fragment patterns with the only exception of *An. punctimacula* (Table 2). However, this species has no geographic overlap with *An. punctipennis* in the USA. Thus, NaeI-based RFLP approach should be applicable for the identification of *An. punctipennis* in different populations throughout the USA.

**Table 2:** Predicted sizes of ITS2 PCR products and patterns of restriction fragments in American species of *Anopheles* based on GenBank data available at [34]

Species	Predicted PCR product size (bp)	Predicted RFLP pattern
*An. punctipennis*	451	283, 168
*An. aquasalis*	532	356, 176
*An. crucians* B	1056	731, 145, 93, 87
*An. darlingi*	592	403, 189
*An. neivai*	490	275, 215
*An. pseudopunctipennis*	555	380, 175
*An. punctimacula*	445	272, 173

### *Anopheles* species distribution in the eastern and midwestern USA

In addition to the development of a tool for molecular diagnosis of *An. punctipennis,* the species composition was analysed in all locations from the eastern and midwestern United States (Table 1, Fig. 1). In the Midwest, *An. punctipennis* was found in Minnetrista, Minnesota where ~ 8% of the specimens were represented by *An. punctipennis* and nearly 92% were *An. quadrimaculatus.* This location is indicated on the map as KW (Fig. 1a). Additional samples from the Midwest were collected in Iowa near Des Moines (IO). In contrast, this collection included 94% of *An. punctipennis* and 6% of *An. quadrimaculatus.*

Another collection set was made in the Appalachian Mountains in Virginia, with elevations ranging from 600 m to 1.2 km (Fig. 1b). Collections in Virginia were made in 3 different environments: town, village, and forest. The town collection was made at the Duck Pond (DP) on the Virginia Tech campus, which is in the center of a small college town of Blacksburg, Virginia. In this setting, 94% of the mosquitoes were *An. punctipennis* with a high population density. The village collection was made in a rural setting in nearby Pandapas Pond (P, PP) in Montgomery County, Virginia. This location contained farms and wildlife. The proportion of *An. punctipennis* in this location reached 65% of the samples collected. The rest was represented by 22% of *An. quadrimaculatus*, 3.7% of *An.* *crucians* C*,* and 5.6% of each of *An.* *crucians* D and *An.* *crucians* E. The third collection was made in a forest location at the high elevation of 1.2 km in Giles County, Virginia near Mountain Lake (MM). In this location, 100% of the mosquitoes were *An. punctipennis,* again with a higher population density than in rural location.

The next set of collections were made in Florida along the coast. Mosquitoes were collected in Western Florida near Panama City Beach and in Eastern Florida near Orlando in urban, rural, and forest locations. *An. punctipennis* was found in only one urban location in Defuniak Springs, Florida in Bruce Creak (BC). The collection consisted of approximately 73% of *An. punctipennis*, 23% of *An. crucians* A*,* 2% of *An. crucians* C*,* and 2% of *An. crucians* E*.* In the coastal areas, the prevalent mosquitoes were from the *An. crucians* complex. In the mineral springs in Panacea, Florida (PF) the sample collection included 92% *Anopheles bradleyi* and 8% *An. quadrimaculatus*. In the more urban setting along the coast, Carrebelle, Florida (CF), approximately 39% *An. crucians* C and approximately 61% *An. crucians* D were found*.* Finally, in the remote forest settings of Apalachicola National Forest in Apalachicola, Florida (SR), 100% of the collection was *An. crucians D.*

### Phylogenetic analysis based on Internal Transcribed Spacer 2

**Fig. 3:**
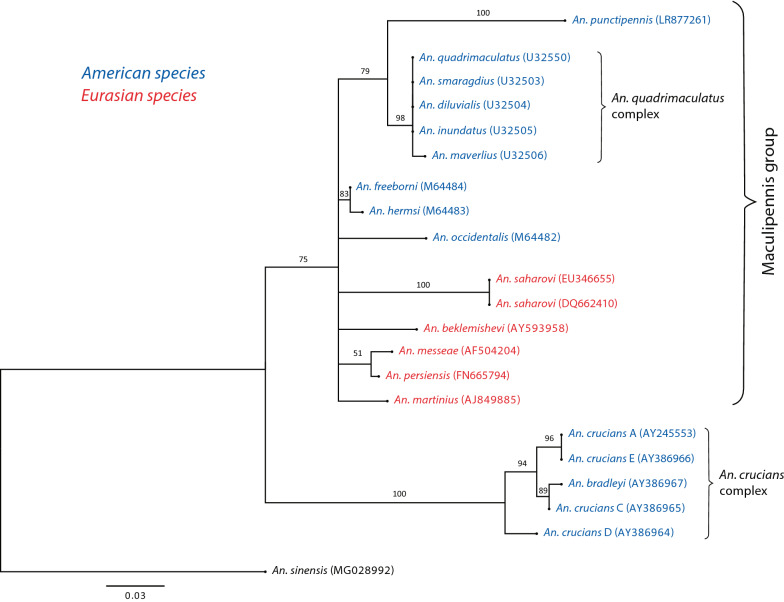
Maximum-likelihood tree based on the ITS2 sequences. Bootstrap values are shown for the branches with support higher than 50%. Branches with bootstrap support less than 50% were collapsed. The phylogenetic analysis placed *An. punctipennis* within the Maculipennis group species

Phylogenetic analyses based on ITS2 sequences using the maximum-likelihood approach [38] allowed us to construct a reliable tree of the major phylogenetic subgroups of the studied species (Fig. 3). The analysis included 20 species: An.punctipennis; 8 North American species from the Maculipennis group (Anophelesfreeborni, Anopheles hermsi, Anopheles occidentalis, and 5 species from An.quadrimaculatus complex); 5 Eurasian species (Anopheles beklemishevi, Anophelesmartinius, Anopheles messeae, Anopheles persiensis, and Anopheles sacharovi), and an outgroup Asian species, Anopheles sinensis (Table 3). Overall, the tree separated all species into two large groups corresponding to species from the Maculipennis group together with *An. punctipennis* and species from the *An. crucians* complex with strong bootstrap support. Within the Maculipennis clade sequences from *An. punctipennis* formed a separate sister clade to *An. quadrimaculatus* with high bootstrap support (79%). Thus, the phylogenetic placement of *An. punctipennis* is unambiguously inside the Maculipennis group according to the analysis of ITS2 sequences.

**Table 3:** Accession numbers and corresponding species names of the ITS2 sequences used for the phylogenetic analysis. The ITS2 sequences are available from the GenBank [30]

Acession number	Species
MG028992	*An. sinensis*
LR877261	*An. punctipennis*
AY593958	*An. beklemishivi*
M64484	*An. freeborni*
M64483	*An. hermsi*
M64482	*An. occidentalis*
AF504204	*An. messeae*
FN665794	*An. persiensis*
AJ849885	*An. martinius*
EU346655	*An. sacharovi*
DQ662410	*An. sacharovi*
U32550	*An. qudrimaculatus*
U32503	*An. smaragdius* (*An. quadimaculatus* B)
U32504	*An. diluvialis* (*An. quadimaculatus* C1)
U32505	*An. inundatus* (*An. quadimaculatus* C2)
U32506	*An. maverlius* (*An. quadimaculatus* D)
AY245553	*An. cricians* A
AY386966	*An. cricians* E
AY386967	*An. bradleyi*
AY386965	*An. cricians* C
AY386964	*An. cricians* D

## Discussion

Among other malaria mosquito species, *An. punctipennis* has an extensive geographic distribution throughout North America [35] and can transmit two important species of human malaria, *P. falciparum* and *P. vivax,* the ungulate malaria parasite *P. odocoilei,* and viruses [10]. Despite these facts, *An. punctipennis* represents one of the most understudied and neglected vectors of malaria in North America, in particular because in the USA malaria was declared eradicated and the major research projects from the last two decades have focused on African malaria mosquito species [2]. Moreover, a molecular diagnostic tool based on ITS2 sequences, the most reliable species identification tool for malaria mosquitoes [15], had never been developed for *An. punctipennis* prior to current investigation. In this study, ITS2 fragments of rDNA were sequenced from 494 field collected mosquito samples in the eastern and midwestern USA. Among them, 276 samples were identified as *An. punctipennis* based on morphological characteristics or COI sequences available at NCBI [34]. All of the *An. punctipennis* samples had a consistent ITS2 sequence length and there was no indication of interindividual variations. Thus, this study determined that *An. punctipennis* represents a single species in the eastern and midwestern states in the USA. However, samples from California and other western states were not included in this study, which requires further attention in the future.

In addition, the RFLP approach based on ITS2 sequences was optimized for 7 species of malaria mosquitoes that were available through current mosquito collections (Table 1). The presence of a unique site in *An. punctipennis* ITS2 sequences for the restriction enzyme NaeI allowed to cleave the PCR product of the ITS2 into two fragments that could be easily identified by gel-electrophoresis. Based on ITS2 sequence comparison available through GenBank [34], only six North American species with NaeI restriction site were identified (Table 2). However, among these species only two *An. crucians B*, *An. pseudopunctipennis* have a geographical overlap with *An. punctipennis* in the USA [39]. Moreover, most of these six species have different sizes of PCR products and RFLP fragment patterns. Thus, this method represents a simple and robust technique for identifying *An. punctipennis* among other local species of *Anopheles* in the USA. The approach also reduces the time and cost of molecular identification and represents a robust method for species identification in field collected mosquitoes.

In current survey, Anopheles mosquito collections were generated from samples collected at 11 locations within 4 states (Florida, Iowa, Minnesota, and Virginia) from urban, rural, and forest settings. *An. punctipennis* was found in 6 locations; only the coastal collection sites in Florida had no *An. punctipennis* samples (Fig. 1). Thus, the geographical distribution analysis showed that *An. punctipennis* is a very abundant species of *Anopheles* in the eastern and midwestern USA. Despite the fact that *An. punctipennis* was considered as a woodland mosquito [35], this species was detected in all of the settings that were examined (i.e., urban, rural, or forest), suggesting a possible broad role of this mosquito in transmission of pathogens. Interestingly, the presence of *An. quadrimaculatus* was determined at high altitude in the Appalachian Mountains (Fig. 1), where the presence of this species had not previously been determined [35]. *Anopheles bradleyi,* which was considered a brackish water mosquito [35], in current study, was found only in Mineral Springs in coastal area of Florida supporting the previous observations.

Finally, the present investigation sheds some light on the phylogenetic relationships of *An. punctipennis* and its systematic position within other species from the genus Anopheles. *Anopheles puctipennis* was a former member of the *An. maculipennis* complex [27], but later was excluded from the complex based on morphological characteristics. According to Harbach [29], *An. punctipennis* belongs to a separate Punctipennis group that includes *An. punctipennis, An. perplexens,* and the *An. crucians* complex. Because of the absence of ITS2 sequences, *An. punctipennis* was not included in the phylogenetic analysis that was conducted for other species from the Maculipennis group [31, 40, 41]. The only phylogeny that included *An. punctipennis* was based on sequencing the D2 region of rDNA [42]. This analysis clearly separated the *An. quadrimaculatus* clade from the *An. freeborni—An. occidentalis—An. earlei—An. punctipennis* clade and suggested *Anopheles walkeri* as the most basal species for the entire group. However, the branches within these two clades remained poorly resolved.

For the better understanding the systematics position of *An. punctipennis,* phylogenetic analysis was performed for 20 species based on ITS2 sequences available from this work and from the GenBank [34]. In addition to *An. punctipennis,* the study included 13 species from the Maculipennis group, 5 species from the *An. crucians* complex, and an outgroup species, *An. sinensis.* The Maculipennis species were selected based on their systematics positions from two North American subgroups, Freeborni (*An. freeborni, An hermsi* and *An. occidentalis*) and Quadrimaculatus (*Anopheles diluvialis, Anopheles smaragdius, Anopheles inundatus, Anopheles maverlius* and *An. quadimaculatus*), and one Eurasian Maculipennis subgroup (*An. beklemishevi, An. martinius, An. messeae, An. persiensis* and *An. sacharovi*). The systematics species statuses of *An. hermsi* [24], species from Quadrimaculatus complex [25, 43], and *An. persiensis* [31] were previously determined based on ITS2 sequencing. The species were also chosen based on their geographic distributions: in western (Freeborni subgroup) and eastern (Quadrimaculatus subgroup) parts in North America [6]; and in northern (*An. beklemishevi,* and *An. messeae*) and southern (*An. persiensis*, *An, martinius,* and *An. sacharovi*) parts of Eurasia [44–46]. Some of the species from *An. crucians* complex (*An. crucians* C, D, and E) included in this study were also identified based on ITS2 sequences [26]. These species are mostly found in eastern parts of North America. An outgroup species *An. sinensis* is a dominant malaria vector in Asia [44]. The maximum-likelihood tree, which was developed here, clearly separated the Maculipennis species from the *An. crucians* complex species and placed *An. punctipennis* as a sister taxon to *An. quadrimaculatus.* Thus, the phylogenetic analysis based on ITS2 sequences allowed us to reliably identify that *An. punctipennis* represents a species within the Maculipennis group and is a close relative to *An. quadrimaculatus*. Further analysis based on multiple genes or the entire genome will provide more details about the phylogenetic relationships of *An. punctipennis* and clarify its systematic position within the Maculipennis group of malaria mosquitoes.

## Conclusions

This study reports the development of a new molecular diagnostic tool for *An. punctipennis*, one of the most widely spread species of malaria mosquitoes in North America. Two versions of the tool, based on either sequence analysis or a cost-effective RFLP approach, were optimized based on field collected material from the eastern and midwestern US. Sequence analysis demonstrated no variations in length or nucleotide polymorphism within the studied samples suggesting that *An. punctipennis* represents a single species in the area of study. The phylogenetic analysis based on ITS2 sequences placed *An. punctipennis* within the Maculipennis group species and separate from species within the *An. crucians* complex.

## Data Availability

The data of ITS2 sequences for *An. punctipennis* are available at NCBI [34] under accession numbers MW581372-MW581485. The consensus sequence is available under accession number LR877261.

## Abbreviations

DNA: Deoxyribonucleic acid
ITS2: Internal transcribe spacer 2
PCR: Polymerase chain reaction
RFLP: Restriction fragment length polymorphism
COI: Cytochrome c oxidase subunit I
rDNA: Ribosomal DNA
NCBI: National Center for Biotechnology Information
BLAST: Basic Logical Alignment Search Tool
